# A structure-function approach to optimizing TLR4 ligands for human vaccines

**DOI:** 10.1038/cti.2016.63

**Published:** 2016-11-02

**Authors:** Darrick Carter, Christopher B Fox, Tracey A Day, Jeffrey A Guderian, Hong Liang, Tom Rolf, Julie Vergara, Zachary K Sagawa, Greg Ireton, Mark T Orr, Anthony Desbien, Malcolm S Duthie, Rhea N Coler, Steven G Reed

**Affiliations:** 1 Infectious Disease Research Institute (IDRI), Seattle, WA, USA; 2 PAI Life Sciences, Seattle, WA, USA; 3 Department of Medicine and Global Health, University of Washington, Seattle, WA, USA

## Abstract

Adjuvants are combined with vaccine antigens to enhance and modify immune responses, and have historically been primarily crude, undefined entities. Introducing toll-like receptor (TLR) ligands has led to a new generation of adjuvants, with TLR4 ligands being the most extensively used in human vaccines. The TLR4 crystal structures demonstrate extensive contact with their ligands and provide clues as to how they discriminate a broad array of molecules and activate or attenuate innate, as well as adaptive, responses resulting from these interactions. Leveraging this discerning ability, we made subtle chemical alterations to the structure of a synthetic monophosphoryl lipid-A molecule to produce SLA, a designer TLR4 ligand that had a number of desirable adjuvant effects. The SLA molecule stimulated human TLR4 and induced Th1 biasing cytokines and chemokines. On human cells, the activity of SLA plateaued at lower concentrations than the lipid A comparator, and induced cytokine profiles distinct from other known TLR4 agonists, indicating the potential for superior adjuvant performance. SLA was formulated in an oil-in-water emulsion, producing an adjuvant that elicited potent Th1-biased adaptive responses. This was verified using a recombinant *Leishmania* vaccine antigen, first in mice, then in a clinical study in which the antigen-specific Th1-biased responses observed in mice were recapitulated in humans. These results demonstrated that using structure-based approaches one can predictably design and produce modern adjuvant formulations for safe and effective human vaccines.

There are two widely recognized phases of the immune response to invading pathogens: the innate response that protects the host rapidly and non-specifically and an adaptive response that is specific for components unique to the pathogen and produce an anamnestic long-lasting, protective response.^1^ Innate immunity is conferred primarily through pattern-recognition receptors triggered by microbe-associated molecular patterns.^2^ This system effectively combines two disparate needs for a receptor–ligand interaction. It has broad enough recognition to respond to an array of diverse patterns on a wide variety of invading organisms; but is specific enough not to respond to similar molecules within the host organism. The elegance of the system is that the molecular patterns that are recognized generally are indispensable for the survival of the invading organism but not found in the host's molecular repertoire.

Triggering the innate system leads to rapid release of cytokines and chemokines in a finely tuned composition that allows the appropriate effector cells to attempt to quell the initial insult from the microbe. At the same time, cells from the adaptive immune system are recruited and trained through presentation of antigens in the context of proper signaling molecules in the draining lymph node. The importance of innate signaling upon expansion of T-cell populations, effective formation of germinal centers, and accelerated hypermutation of B cells is becoming clear from studies in which antigens were presented with or without added innate stimulation provided by adjuvants.^3, 4^

Many of the toll-like receptors (TLRs) are being targeted for adjuvant development, but TLR4 is especially attractive as there is a very large human safety database supporting the use of TLR4 ligands in prophylactic vaccines (for example, Cervarix), the favorable receptor distribution (TLR4 is expressed only on differentiated, non-dividing antigen-presenting cells in humans, unlike other TLRs) and preserved expression as the immune system ages, hold promise for vaccines to protect the very young as well as the elderly.^5^ The TLR4 receptor dimerizes with the myeloid differentiation factor MD2 and then this complex homodimerizes when fully assembled.^6^ TLR4 ligands can be loaded through helper proteins such as the lipopolysaccharide (LPS)-binding protein which shifts the agonist to CD14 that then can diffuse on the membrane and load the TLR4/MD2 complex.^7^ MPL is a TLR4 ligand originally developed by Ribi ImmunoChem Research Inc (Hamilton, MT, USA), and then by Corixa Corporation (Seattle, WA, USA), that is now being used in adjuvants produced by GlaxoSmithKline (Rixensart, Belgium) and is the only TLR ligand specifically added to commercial vaccines.^8, 9, 10^ MPL is a purified natural product derived from *Salmonella minnesota* containing a mixture of molecular species with different acyl chain lengths, numbers and affinities for mammalian TLR4. When used in the context of the cervical cancer vaccine Cervarix it has been shown to enhance and broaden the immune response as well as providing long-term immunity in humans.^11, 12^ The molecule glucopyranosyl lipid adjuvant (GLA) is a synthetic TLR4 agonist that has similarity to a hexacylated component of MPL and was a first-generation molecule formulated under Current Good Manufacturing Practice regulations by our group for testing in human clinical trials.^13^

GLA was produced on the basis of similarity to natural compounds and not by inspection of the known receptor/ligand interactions of TLR4. In this paper we report using the known structures for TLR4/MD2 to design a new agonist, Second-generation lipid adjuvant (SLA) targeted at the human receptor. The crystal structure of the complex^6^ guided a shortening of the lipid chains to develop a targeted molecule made for human-receptor agonism. The new molecule was synthesized and tested in cell lines and primary human cells and then—formulated in an oil/water emulsion—used in preclinical models to verify adjuvant activity. This was followed by a phase 1 clinical study in healthy adults in the US to test if the designer molecule had agonist properties comparable to or distinct from the structurally related GLA delivered in the same formulation. As predicted by the *ex vivo* and preclinical studies, formulated SLA displayed promise as a safe and effective modern designer adjuvant, eliciting potent and directed immune responses in human subjects.

## Results

### Design of a second-generation TLR4 ligand, SLA

**Figure 1:**
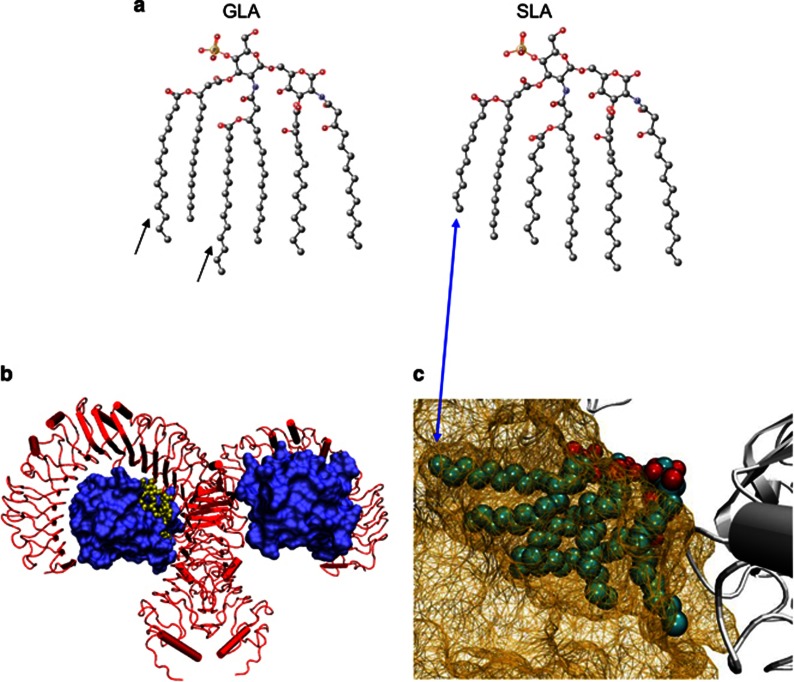
**Structural design of SLA.** (**a**) Structural modifications introduced in SLA. Small black arrows under the molecule ‘GLA' indicate where the GLA molecule was truncated to form SLA. By eliminating carbons at the end of GLA's acyl chains, SLA should have a better fit into MD2, allowing a more compact interaction with the TLR4/MD2 dimer (blue double-sided arrow). (**b**) TLR4/MD2 crystal structure. The structure of the human TLR4 (red)/MD2 (blue) homodimer of heterodimers is shown. (**c**) Enlarged view of the TLR4/MD2 structure focusing on the acyl chains in the MD2 pocket. In the crystal structure the MD2 pocket envelops the acyl chains but only accommodates a certain volume. The excess volume causes the disaccharide head group to stick out further into the interstitial space between TLR4 and MD2. The shorter chains on SLA are predicted to fit better into this pocket (blue double-sided arrow).

Investigation of the hydrophobic pocket of the MD2/TLR4 complex (Figure 1b) suggested that there are energy penalties to crowding it with large lipid chains. A lipid-A homolog with such long acyl groups would likely still bind, but at the cost of the head group extending out of MD2, up toward the TLR4 dimerization partner potentially causing conformational changes and/or low affinity binding. We targeted the more limited space that in the human TLR4 crystal structure accommodates the lipid chains hanging from the disaccharide moiety of LPS by removing methylene groups from extended chains (Figure 1a). These are present in GLA and provide a jagged-end surface on a 2D projection (Figure 1a). Removing these four carbons from each of the extending acyl chains maintains as much hydrophobic surface area as possible to provide favorable binding energetics (Figure 1c). On the basis of the hypothesis that these changes would result in a molecule with improved affinities and better signaling properties this designer candidate was produced synthetically.

### Biophysical characteristics of adjuvant formulations of SLA

SLA was formulated as an aqueous, micellar suspension of the molecule that is stable and suitable for use in cell assays (aqueous formulation (AF); ‘SLA-AF'). The size characteristics of SLA-AF appeared similar to those of GLA-AF (Supplementary Table 1), with an intensity-based average size of 80 nm. As with GLA-AF, the intensity-based average size was biased toward a population of larger particles in the formulation; a volume-based size distribution of SLA-AF or GLA-AF revealed a majority of smaller particles comprising the size distribution of the formulation (Supplementary Figure 1). For preclinical *in vivo* testing, SLA was also formulated into a squalene-based oil-in-water emulsion (‘SLA-SE') of ~90 nm size droplets with low polydispersity, indicating unimodal size distribution verifying that differences of the adjuvant formulations are not due to intrinsic differences in the biophysics of the formulation, but rather due to differences in the signaling properties of the incorporated agonist (Supplementary Figure 1 and Supplementary Table 1).^14^

### *In vitro* activity of SLA on cell lines

**Figure 2:**
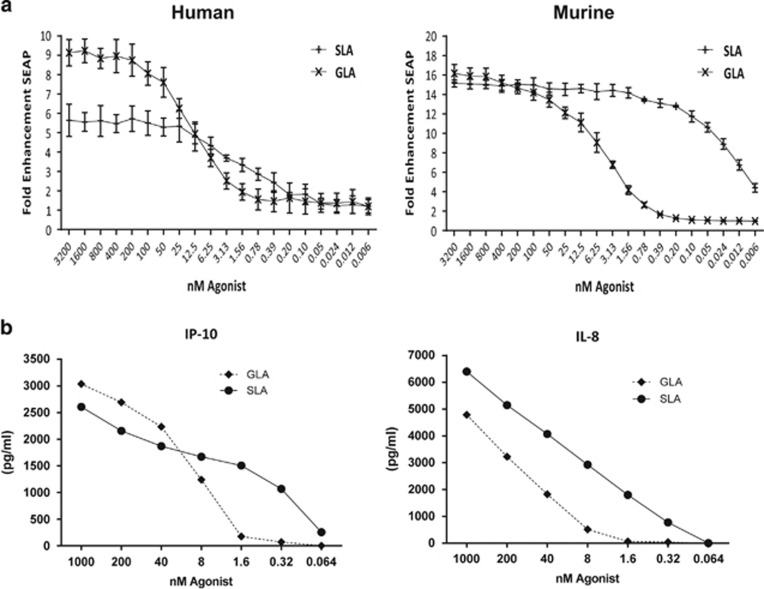
**Biological activity comparison of TLR4 ligands on cell lines.** (**a**) Human and murine TLR4/MD2 transfected cell lines were used to measure fold NF-κB expression. Both human- and murine-based lines show a trend for SLA being more potent with an earlier rise of NF-κB signal and a close to 500-fold difference in the murine line (experiment was performed independently twice, three replicate points averaged). (**b**) Mono Mac 6 cells were stimulated with either GLA or SLA in an aqueous formulation. This human cell line mirrored the increased sensitivity to stimulation with SLA by producing cytokines at lower levels than those required for signal using GLA.

To verify that SLA signaled through TLR4 and to determine if there were significant differences in the profile of AF of SLA versus GLA *in vitro*, transfected HEK cells expressing either murine or human TLR4/MD2 were stimulated with serial dilutions of the respective ligand formulated as a micelle in the AF. In this assay, SLA-AF caused a more rapid rise in reporter signaling than GLA-AF and plateaued at a lower concentration in cells expressing the human TLR4 dimer (Figure 2a, left panel ‘human'). The GLA-AF formulation reached its 50% effective dose at 3.13 nM in cells transfected with the murine TLR4, whereas the SLA-AF formulation in this system reached this 50% level at a roughly 250-fold lower concentration of 0.012 nM (Figure 2a, right panel ‘murine'). No activity was seen when similar, TLR2 transfected, cell lines were stimulated with the ligand as an AF formulation, whereas these cells responded strongly to natural ligand (Supplementary Figure 2) confirming specificity of TLR4 signaling of the agonist and no additional TLR2 signaling as has been reported for some LPS preparations. Similar to the reporter cell line, Mono Mac cells, a human cell line that has features of mature monocytes which respond *in vivo* to TLR4, responded to strongly to stimulation with SLA-AF (Figure 2b).

### *In vitro* activity of SLA on primary human cells

**Figure 3:**
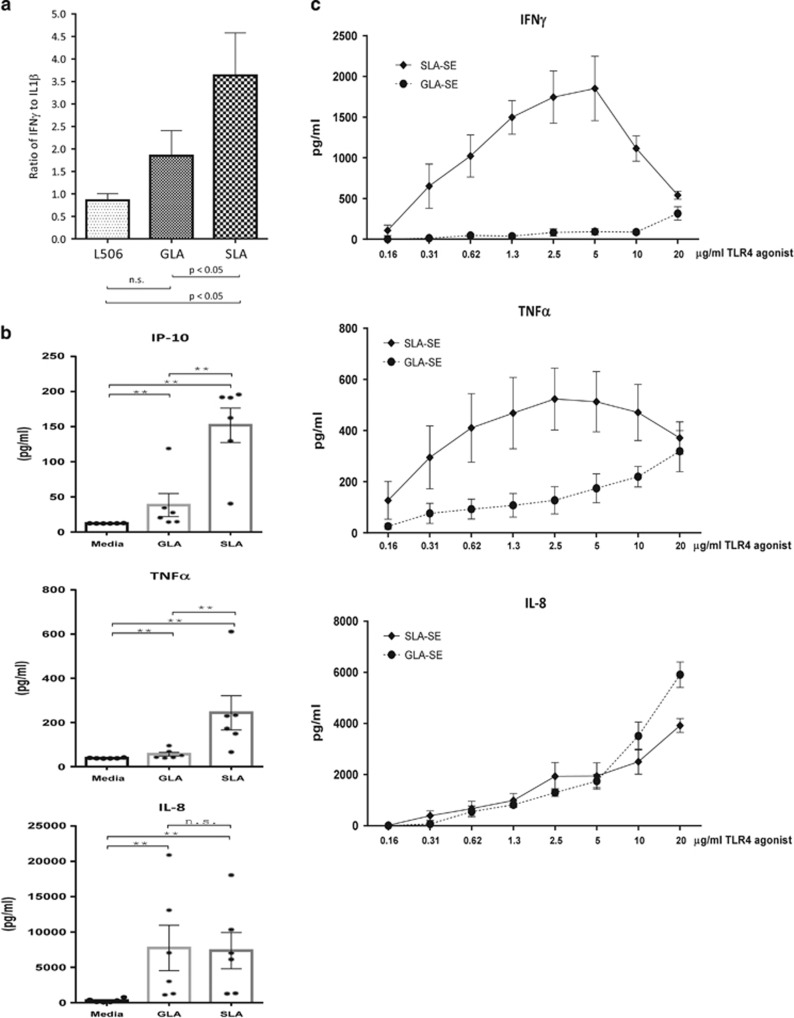
**Biological activity comparison of TLR4 ligands on primary cells.** (**a**) Cytokine release from human whole blood. As shown in the graph, SLA-AF significantly enhances the amount of IFNγ produced per unit of IL1 β, a hallmark of inflammation. Statistically significant differences between indicated groups were determined by analysis of variance with Bonferroni's correction for multiple tests, NS indicates not significant. (**b**) Cytokine release from human dendritic cells. At 1 nM agonist SLA-AF induced 100 pg to low ng levels of cytokines and chemokines. (**c**) Formulated SLA strongly stimulates human cells. When formulated as a squalene emulsion, SLA-SE strongly stimulates human whole blood *in vitro*.

To examine if the qualitative differences observed in cell lines were recapitulated in primary cells, we also tested whether formulated SLA elicited a unique cytokine profile in whole blood and from monocyte-derived dendritic cells. As with the cell lines, SLA formulations appeared to induce a different cytokine profile in primary cells when compared with the related molecule GLA.^15^ Figure 3a shows that per unit of the inflammatory cytokine IL-1b, SLA elicited the production of significantly more IFNγ. These pattern differences held for numerous responses to whole-blood stimulation with either SLA-AF or GLA-AF, although as seen in Figures 2 and 3, the differences are not seen across all cytokines (for example, IL-8). The general profile in this system is more Th1 biasing with less IL-10 family members being secreted while maintaining the levels of IFNγ, IFNα, IL12p70 and IP-10 (Supplementary Figure 3). Human dendritic cells were quite sensitive to stimulation with SLA-AF and 1 nM concentrations of this molecule were sufficient to induce cytokine and chemokine secretion up to low ng levels consistent with the ability to evoke Th1 biasing innate immunity (Figure 3b). When the molecule was then formulated to produce a combination adjuvant of a squalene-based inflammasome stimulant SE with SLA similar to that reported in,^16^ primary cells responded strongly to stimulation with this SLA-SE adjuvant, an indicator that the formulation would be quite potent in humans (Figure 3c).

### *In vivo* activity of formulated SLA in mice and humans

**Figure 4:**
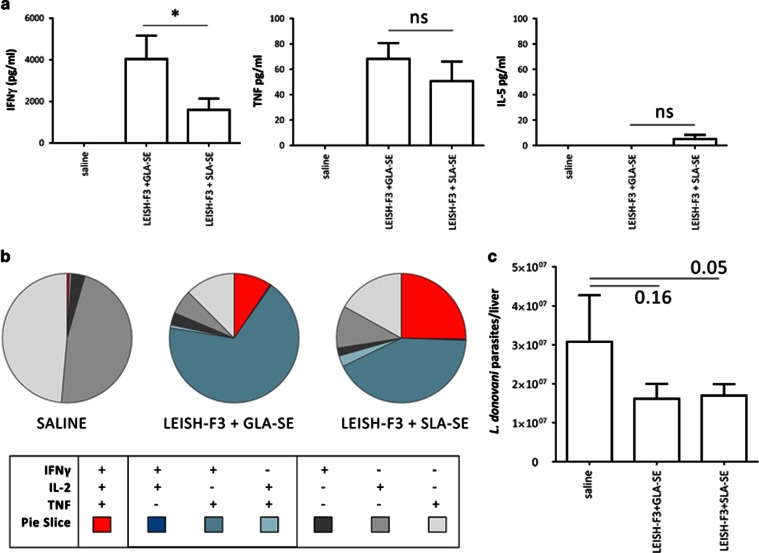
**SLA is a functional adjuvant in mice.** (**a**) Cytokine responses to LEISH-F3. Mice were immunized and spleen cells incubated with LEISH-F3 antigen. IFNγ secreted into the culture supernatant was measured by enzyme-linked immunosorbant assay. (**b**) Cellular responses to LEISH-F3—cells were subjected to flow cytometry and CD4 T cells were categorized on the basis of producing three, two or one cytokine(s) (IFNγ, IL-2 and/or TNF). Three mice per group were evaluated. Student's *t*-tests were used for statistical analysis. (**c**) Protection against parasite challenge in mice *in vivo*. Immunized mice were inoculated by intravenous infection with *L. donovani* parasites and burden in the liver 4 weeks later determined by PCR. Seven mice per group were evaluated. Statistically significant differences between indicated groups were determined by analysis of variance with Bonferroni's correction for multiple tests, NS indicates not significant. Results are representative of at least two independent experiments.

To assess whether SLA-SE functioned as a potent adjuvant *in vivo* when administered with a recombinant protein, mice were immunized with the LEISH-F3 antigen formulated either in GLA-SE or in SLA-SE and we evaluated the immune responses that were induced following immunization. Previously, we had shown that the LEISH-F3 antigen is dependent on adjuvant to induce Th1 responses and protection.^17^ As expected, significant quantities of antigen-specific antibodies were induced when mice were immunized with antigen in conjunction with GLA-SE or SLA-SE (Supplementary Figure 4). When spleen cells from immunized mice were incubated with antigen, large quantities of IFNγ and TNF, but not IL-5, were secreted (Figure 4a). Approximately three quarters of the antigen-specific CD4 T cells in mice immunized with LEISH-F3/GLA-SE or LEISH-F3/SLA-SE had a polyfunctional Th1 phenotype, producing double and triple combinations of IFNγ, IL-2 and TNF (Figure 4b). Furthermore, mice immunized with the LEISH-F3 antigen formulated in GLA-SE or SLA-SE exhibited lower parasite burdens after challenge by intravenous *Leishmania donovani* infection than mice that were not immunized (Figure 4c).

**Figure 5:**
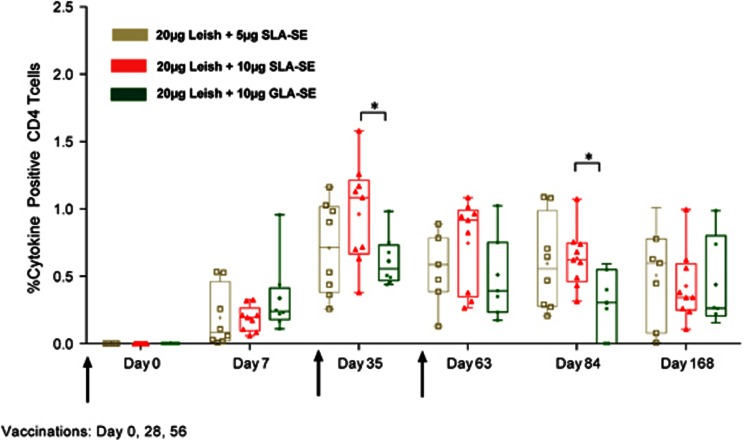
**SLA-SE stimulates potent responses in humans.**Subjects were immunized with the indicated formulations three times, at specified intervals, for the analysis of LEISH-F3-specific immune responses. Percentages of cytokine-positive CD4^+^ T cells expressing any combination of IFNγ, TNF or IL-2 (*n*=9 or *n*=8). Box and whisker min to max indicates 25th to 75th percentiles, internal bar indicates the median, plus sign indicates the mean. Statistics were performed using a two-way analysis of variance with Tukey's multiple comparisons test between cytokine groups at a single time point. *P<0.05.

In the clinical study, antigen-specific immune responses of volunteers who received all three study injections were evaluated. Injection of antigen alone did not generate responses (data not shown), indicating the importance of adjuvant in promoting antigen-specific CD4 T-cell responses. In terms of the cellular responses to LEISH-F3 (Figure 5), the vaccine was immunogenic at both 5 μg and 10 μg of SLA-SE doses. At day 35, that is, 7 days following the second immunization, the percentage of CD4-positive T cells positive for IFNγ, TNF and IL-2 in response to LEISH-F3 antigen stimulation was higher in subjects immunized in the context of 10 μg of SLA-SE than 10 μg GLA-SE. At day 84, the percentage of CD4-positive T cells positive for IFNγ, TNF and IL-2 was still significantly different between these 2 cohorts. Although T-cell responses were still detected at day 168, there was no statistical difference than those measured at days 35 and 84 for IFNγ and TNF. Together, these data indicate that like LEISH-F3+GLA-SE, LEISH-F3+SLA-SE vaccination is safe in humans and induces antigen-specific cellular responses that could protect against *Leishmania* infection.

## Discussion

The recognition of ‘microbe'-associated molecular patterns by the innate immune system is mediated by a class of receptors that combine two disparate features: (1) recognition of a broad array of molecules of a single class and (2) narrow recognition of just that class of molecules. As was shown with the development of other TLR4 agonists like the AGPs,^18^ TLR4 appears to be one of the most broad in terms of agonists that it accepts: they vary by the carbohydrate head groups, the number, position and length of lipid chains attached to the disaccharide core as well as the charge imparted by the 1 and/or 4′ phosphate groups.^19^ There are large areas of contact between ligands and TLR: in the case of TLR4 the lipid chains of the ligand are almost completely encased in the MD2 pocket and the subtle alterations to the lipid A structure reported here resulted in large changes in innate signals that suggest a discriminatory system that can trigger different profiles of pro-inflammatory, regulatory or interferon responses depending on a single receptor–ligand interaction. By leveraging this non-binary, ‘fuzzy', receptor/ligand response pattern one can design stimulatory adjuvant molecules that induce more of the type of immunity desired while separating that activity from the induction of pyrogenic and inflammatory responses. In the present study, we demonstrate for the first time that subtle changes in molecular structure may lead to significant differences in biological activity. This was demonstrated most clearly in studies on cell lines, but also reflected in mouse and human *in vivo* immunogenicity. Thus, synthetic molecules can be designed to improve on naturally occurring structures to achieve desired immunological outcomes.

TLR4 has the unique ability among the TLR family of receptors to signal via TRIF (TIR-domain-containing adaptor-inducing interferon-b) and MyD88.^20, 21^ On the basis of our results, it appears the putative compact SLA binding results in a cytokine profile more reminiscent of TRIF-dependent signaling compared with GLA as it induces less inflammatory cytokines, such as the secreted IL-1β, a characteristic of MyD88 signaling, whereas maintaining Th1 biasing chemokines and cytokines like interferon and IP-10. This is in line with a recent study that also concluded that a TRIF pathway-biased shift could be obtained by including shorter acyl chains in the constructs.^22^ The unique sensitivity of human cells to the designer molecule could then be a combination of higher binding affinities due to less structural collision with the acyl chains combined with the induction of more type 1 interferons due to the TRIF bias.

We had previously reported that combining interferon signaling with caspase dependent signaling via the inflammasome results in powerful adjuvant effects through the combined action of IL18 and interferon.^16^ Combining an agonist with a stronger interferon signal with a carrier that provides inflammasome activation should result in a potent adjuvant. This principal was used to then produce SLA-SE and test it in *in vivo* models. As predicted, the combination adjuvant strongly stimulated human cells and resulted in good pathogen protection in mice as well as yielding potent immune responses in humans.

Although GLA-based adjuvants have proven safe and effective in dozens of past or current clinical studies, the current report suggests that TLR4 ligands can be fine-tuned to optimize immune responses. The ability to rationally design new agonists of the innate immune system is a powerful tool for vaccine and immune therapy developers. Tuning cytokine profiles to minimize potentially deleterious inflammatory responses and keying in on molecules that induce human cytokine profiles favorable for preventing or treating allergies, viral diseases and cancers opens the door to safe, convenient, and effective adjuvant mono-therapies and vaccines. We introduce here a molecule in human clinical development that was built on structural design principles and promises to be a lead candidate for inclusion in second-generation combination adjuvant formulations.

## Methods

### Ligand design and comparison

Ligands were sketched and imported into the Avogadro software package version 1.1.0 (http://avogadro.openmolecules.net/).^23^ Energy minimization was performed using the UFF forcefield and steepest descent algorithm until the change in energy was below 1 kJ per mol. Structures were exported as CML files. The pdb file for human TLR4 with MD2 in the presence of LPS^6^ was downloaded and edited to remove all chains except those representing a single LPS molecule. Images were made with VMD support. VMD is developed with NIH support by the Theoretical and Computational Biophysics group at the Beckman Institute, University of Illinois at Urbana-Champaign.^24, 25, 26^

### Formulation of TLR4 agonists

GLA was obtained as PHAD from Avanti Polar Lipids Inc (Alabaster, AL, USA) or synthesized at Corden Pharma (Liestal, Switzerland). DPPC (1,2-dipalmitoyl-sn-glycero-3-phosphocholine) and egg phosphatidylcholine were purchased from Avanti Polar Lipids (Newark, NJ, USA). DMPC (1,2-dimyristoyl-sn-glycero-3-phosphocholine) was obtained from Avanti Polar Lipids, Lipoid, or NOF America (Irvine, CA, USA). SLA was synthesized by Avanti Polar Lipids, Corden Pharma, or in collaboration with Dr Robert William's lab at Colorado State University. Squalene was purchased from Sigma-Aldrich (St Louis, MO, USA). Poloxamer 188 and glycerol were obtained from Spectrum Chemical (Gardena, CA, USA). Buffer components were purchased from J.T. Baker (San Francisco, CA, USA).

Aqueous suspensions of SLA (SLA-AF) or GLA (GLA-AF) were manufactured by mixing DPPC and the TLR4 ligand at a 2:1 DPPC:SLA molar ratio in chloroform, which was then evaporated. Ultrapure water was added to the resulting dried film, and the mixture was sonicated in a VWR 75D (VWR International, West Chester, PA, USA) or Crest Powersonic CP230D (Crest Ultrasonics, Trenton, NJ, USA) sonicating water bath at ~60 °C until the formulation was translucent.

Oil-in-water emulsion formulations of SLA (SLA-SE) or GLA (GLA-SE) containing squalene were manufactured by high-speed mixing a buffered aqueous phase (containing poloxamer 188, glycerol and ammonium phosphate buffer) and an oil phase (containing squalene, egg phosphatidylcholine or DMPC, and SLA or GLA) at ~7000–10 000 r.p.m. for ~10 min followed by microfluidization for 10–12 passes at 30 000 psi as described previously with minor modifications.^27^

Particle size was measured using the Malvern Instruments (Worcestershire, UK) Zetasizer Nano-S or -ZS via dynamic light scattering. SLA-AF or GLA-AF was diluted 10-fold in water, whereas SLA-SE or GLA-SE was diluted 100-fold in water, in triplicate.

The concentration of SLA or GLA was measured by high-performance liquid chromatography with charged aerosol detection. For SLA-AF or GLA-AF, the formulation was first diluted 20-fold into mobile phase A (75:15:10 (v:v:v) methanol:chloroform:water with 20 mM ammonium acetate and 1% acetic acid). For SLA-SE or GLA-SE, the formulation was first diluted 20-fold into mobile phase B (1:1 (v/v) methanol:chloroform with 20 mM ammonium acetate and 1% acetic acid). Each formulation was assayed in triplicate. All samples were injected on a Waters Co (Milford, MA, USA) Atlantis T3 column attached to an Agilent Model 1100 HPLC (Agilent, Santa Clara, CA, USA). A gradient consisting of mobile phases A and B was employed over 25 min. Detection was performed using an ESA Biosciences (Chelmosford, MA, USA) Corona Charged Aerosol Detector.

### Transfected cell line assay

All compounds were diluted tenfold in phosphate-buffered saline (PBS) to the nM starting concentration, then serially diluted in PBS. Next, 20 μl of each dilution were added in triplicate to a final volume of 200 μl to wells each containing 50 000 HEK-Blue TLR4 cells (Invivogen, San Diego, CA, USA) that expressed either the human TLR4 (Cat. Code hkb-htlr4) or the murine TLR4 gene (Cat. Code hkb-mtlr4). The 96-well plates were then incubated for 24 h at 37 °C/5% CO_2_ in a humidified incubator. In all, 10 μl of supernatant from these stimulated cultures were then added to 90 μl SEAP media (Invivogen) for colorimetric detection of NF-κB reporter-driven secreted alkaline phosphatase activity. Absorbance was then read at 650 nm after 2 h incubation at 37 °C. Signal was divided by that read for the PBS alone control and is reported as ‘fold enhancement'.

### Stimulation of MonoMac6 cells

The human acute monocytic leukemia cell line MonoMac6 was obtained from DSMZ (ACC 124, Braunschweig, Germany), and cultured according to the manufacturer's instructions. All compounds were diluted to 2 μM, and then serially diluted in fivefold steps in culture media. A total of 100 μl containing 1.5 × 10^5^ cells were plated into 96-well flat-bottom plates; and 100 μl of each serial dilution was added to the plated MonoMac6 cells, providing a final well volume of 200 μl with a top compound concentration of 1 μM. Samples were incubated for 24 h at 37 °C/5% CO2 in a humidified incubator and supernatants assayed for select cytokines as described below.

### Stimulation of human whole blood

All human blood research reported here is reviewed and approved by Western Institutional Review Board and all human subjects undergo an IRB-approved informed consent process. For the AF samples, human blood samples were collected from normal, healthy donors using standard phlebotomy techniques. All compounds were diluted to 10 μM, and then serially diluted in fivefold steps in irrigation-grade saline. A total of 100 μl whole blood was then mixed into 100 μl of the agonist diluted in irrigation-grade saline. Samples were incubated for 24 h at 37 °C/5% CO_2_ in a humidified incubator and supernatants assayed for select cytokines as described below. Eight donor mean values were collected in duplicate and plotted as concentration of cytokine.

For the SE formulations, heparinized human blood samples were collected from four normal, healthy donors using standard phlebotomy techniques. All formulations were diluted to 0.2 mg ml^−1^ TLR4 agonist/8% oil, and then serially diluted in twofold steps in irrigation-grade saline. In all, 20 μl of each serial dilution was added to 96-well U-bottom plates, followed by the addition of 180 μl of whole blood, providing a final well volume of 200 μl with a top formulation concentration of 20 μg ml^−1^ TLR4 agonist/0.8% oil. Samples were incubated for 24 h at 37 °C/5% CO_2_ in a humidified incubator and plasma supernatants assayed for select cytokines as described.

### Detection of cytokines

Enzyme-linked immunosorbant assays were used to quantify the indicated cytokines (BD Biosciences, San Jose, CA, USA) in culture supernatants. Absorbances were measured at 450 nm (Molecular Devices, Sunnyvale, CA, USA). Data are presented as means ± s.e.m. of values from duplicate wells.

### Murine immunizations

Female C57BL/6 mice (purchased from Charles River Laboratories, Wilmington, MA, USA) were maintained in specific pathogen-free conditions and in accordance with animal procedures approved by the IDRI institutional animal care and use committee. Mice entered experiments at 6–8 weeks of age and were immunized by subcutaneous injection of the Leish-F3 recombinant protein^17^ formulated with adjuvant at the base of the tail. Vaccines were prepared to provide a total of 5 μg per dose protein and 5 μg per dose GLA-SE or SLA-SE in a total volume of 0.1 ml. Mice were injected a total of three times at three week intervals. One month after the final immunization, spleens were removed and single-cell suspensions prepared. Mononuclear cells were enumerated using a ViaCount assay with a PCA system (Guava Technologies, Hayward, CA, USA). Cells were cultured at 2 × 10^5^ cells per well in duplicate in a 96-well plate (Corning Incorporated, Corning, NY, USA) in RPMI-1640 supplemented with 5% heat-inactivated fetal calf serum and 50 000 Units penicillin/streptomycin (Invitrogen), in the presence of 10 μg ml^−1^ protein. Culture supernatants were harvested after 4 days and cytokine content determined by enzyme-linked immunosorbant assay, according to the manufacturer's instructions (eBioscience, San Diego, CA, USA). To assess CD4 T-cell responses spleen cells were incubated with antigen, then stained for membrane expression of CD4 (clone GK1.5), and CD8 (clone 53–6.7; both BD Biosciences). Cells were then fixed and permeabilized in Cytofix/Cytoperm (BD Biosciences) according to manufacturer's instructions and stained for intracellular expression of IFNγ (clone XMG1.2), IL-2 (clone JES6-5H4) and TNF (clone MP6-XT22; BD Bioscience). Cells were washed and suspended in PBS, then 10^6^ events acquired using a BD LSRFortessa (BD Biosciences). Intracellular molecules were analyzed, after gating through CD4^+^/CD8^−^ T cells, with FlowJo (Tree Star Inc, Ashland, OR, USA), Pestle and SPICE (NIAID, NIH (BCBB)).

### *L. donovani* infection and parasite quantification

*L. donovani* (MHOM/SD/00/1S-2D) were routinely passed through Syrian golden hamsters to generate virulent amastigote and promastigote stocks in M199 medium. Mice were challenged by retro-orbital intravenous injection with either 1 × 10^6^ *L. donovani* parasites. Four weeks after infection, livers were harvested and DNA was extracted from homogenate using QIAmp DNA mini kits (QIAGEN, Hilden, Germany). DNA was quantified using Nanodrop UV-Vis spectrophotometer (ND-1000, Wilmington, DE, USA). *L. donovani* DNA was detected using primers as shown in Supplementary Table 2. Mouse Gapdh FAM (Thermo Fisher Scientific, Waltham, MA, USA) was used as an internal reference control. Cp's of samples were fitted to a standard curve to determine number of parasite per μl of DNA. Final parasite burdens are expressed in number of *Leishmania* parasites per organ.

### Clinical Study

The safety, tolerability and immunogenicity of LEISH-F3+GLA-SE and LEISH-F3+SLA-SE was evaluated in a first-in-man phase 1, randomized, open-label and dose-escalation study. A total of 36 healthy adults (male and female adults with no history of travel to *Leishmania*-endemic areas) were recruited enrolled after signing the obtaining informed consent form, then randomly sequentially assigned to receive injections as follows: 9 subjects were assigned to 20 μg LEISH-F3+5 μg SLA-SE, 9 subjects to 5 μg LEISH-F3+10 μg GLA-SE, 9 subjects to 20 μg LEISH-F3+10 μg GLA-SE and 12 subjects to 20 μg LEISH-F3+10 μg SLA-SE. Study injections were given by intramuscular injection on days 0, 28 and 56; subjects were followed for 1 year following the last injection (through day 421). Blood samples were collected for measurement of hematology and serum chemistry parameters at screening and on days 7, 35 and 63. Vital signs were collected at every study visit and before and 30 min after each study injection on days 0, 28 and 56. Final resulting evaluable groups for PBMC Luminex (against the components of the LEISH-F3 protein, NH and SMT) were: 2 μg GLA-SE (*n*=6), 5 μg GLA-SE (*n*=9) and LEISH-F3 alone (*n*=7).

### Statistical Analyses

Statistically significant differences between groups in the whole-blood data and the animal experiments were determined by pairwise analysis of variance comparisons with Bonferroni's correction for multiple tests using GraphPad Prism version 6.00 for Windows (GraphPad Software, La Jolla, CA, USA; www.graphpad.com.
