# Supplementary material for: A structure-function approach to optimizing TLR4 ligands for human vaccines
Source: Clin Transl Immunology. 2016 Nov 2;5(11):e108–. doi: 10.1038/cti.2016.63 (PMC5133366; doi:10.1038/cti.2016.63)
Supplement: Supplementary Information [file cti201663x1.doc]

**Supplemental** Table 1: Size characteristics of formulations of SLA and GLA

| **Formulation Name**  **(number manufactured*)** | **Manufacturing Method** | **Particle Size**  **(Z-ave, nm)** | **Size Polydispersity**  **(PdI)** |
| --- | --- | --- | --- |
| SLA-AF (n=7) | Sonication | 84.4 (±19.3) | 0.234 (±0.016) |
| GLA-AF (n=3) | Sonication | 84.5 (±15.1) | 0.267 (±0.008) |
| SLA-SE (n=3) | Microfluidization | 85.9 (±7.1) | 0.048 (±0.005) |
| GLA-SE (n=3) | Microfluidization | 82.7 (±4.5) | 0.048 (±0.005) |

*Formulations were manufactured at SLA and GLA concentrations ranging from 0.2 to 1 mg/ml

**Supplemental Table 2: Primers used for PCR**

| **Primer target** | **Primer Sequence** | **Comment** |
| --- | --- | --- |
| L42486 forward | 5’- GCGACGTCCGTGGAAAGAA-3’ |  |
| L42486 reverse | 5’ -GGCGGGTACACATTAGCAGAA-3’ |  |
| FAM reporter | 5’- CAACGCGTATTCCC-3’ | Detects a 203-bp genomic repeat region specific to *Leishmania* species (NCBI Blastn). |

**Supplemental Figure 1. Particle Size of SLA formulations.**


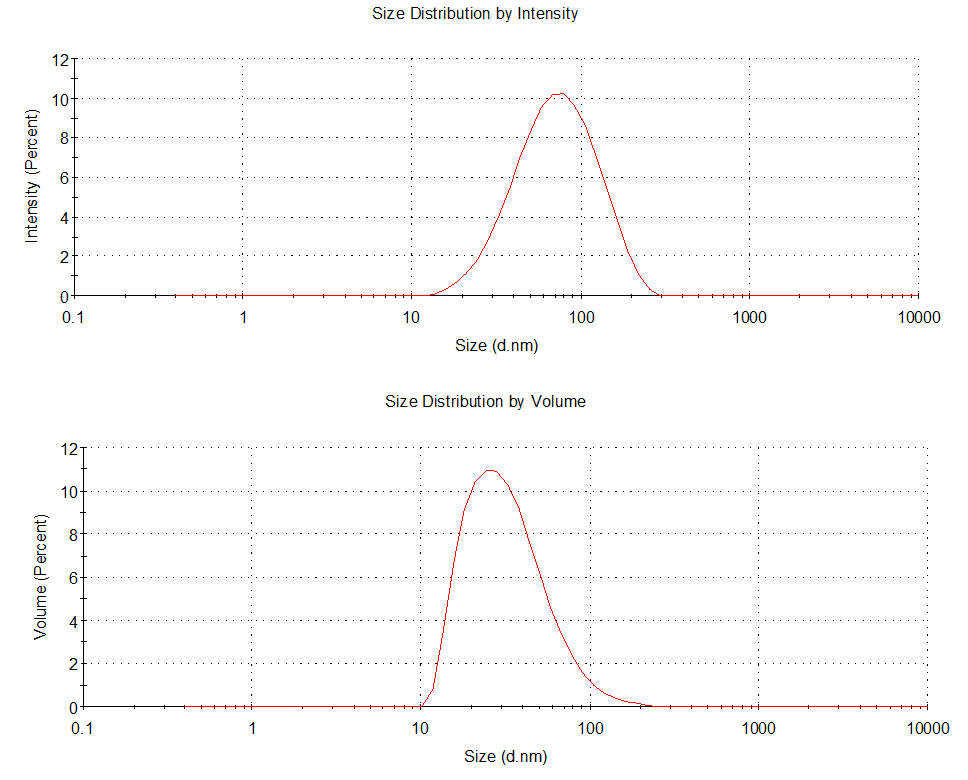


**Supplemental Figure 2. TLR2 signaling is not induced by SLA.**

**Supplemental Figure 1. Biological activity comparison of TLR4 ligands on primary cells**

**
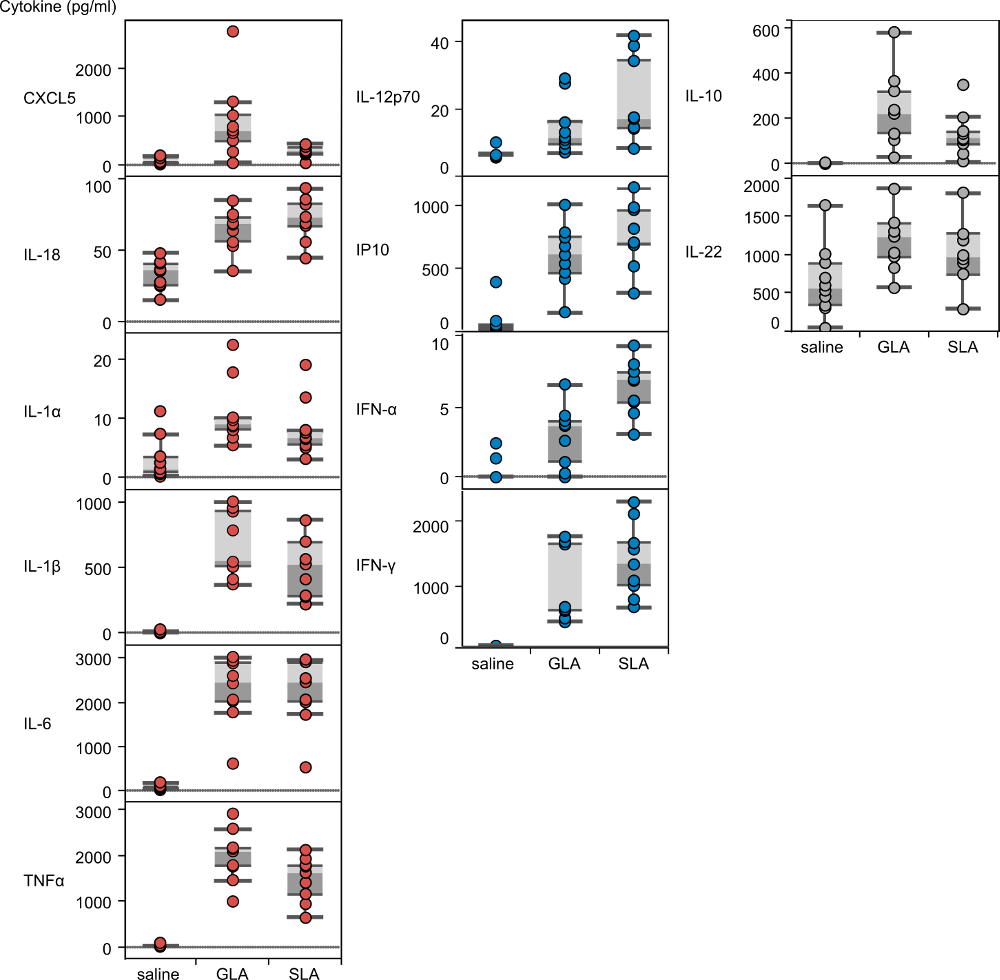
**

**IFN**

**IL-10**

**Inflammatory**

**Supplemental Figure 4. Antibody responses to vaccination in mice**

**
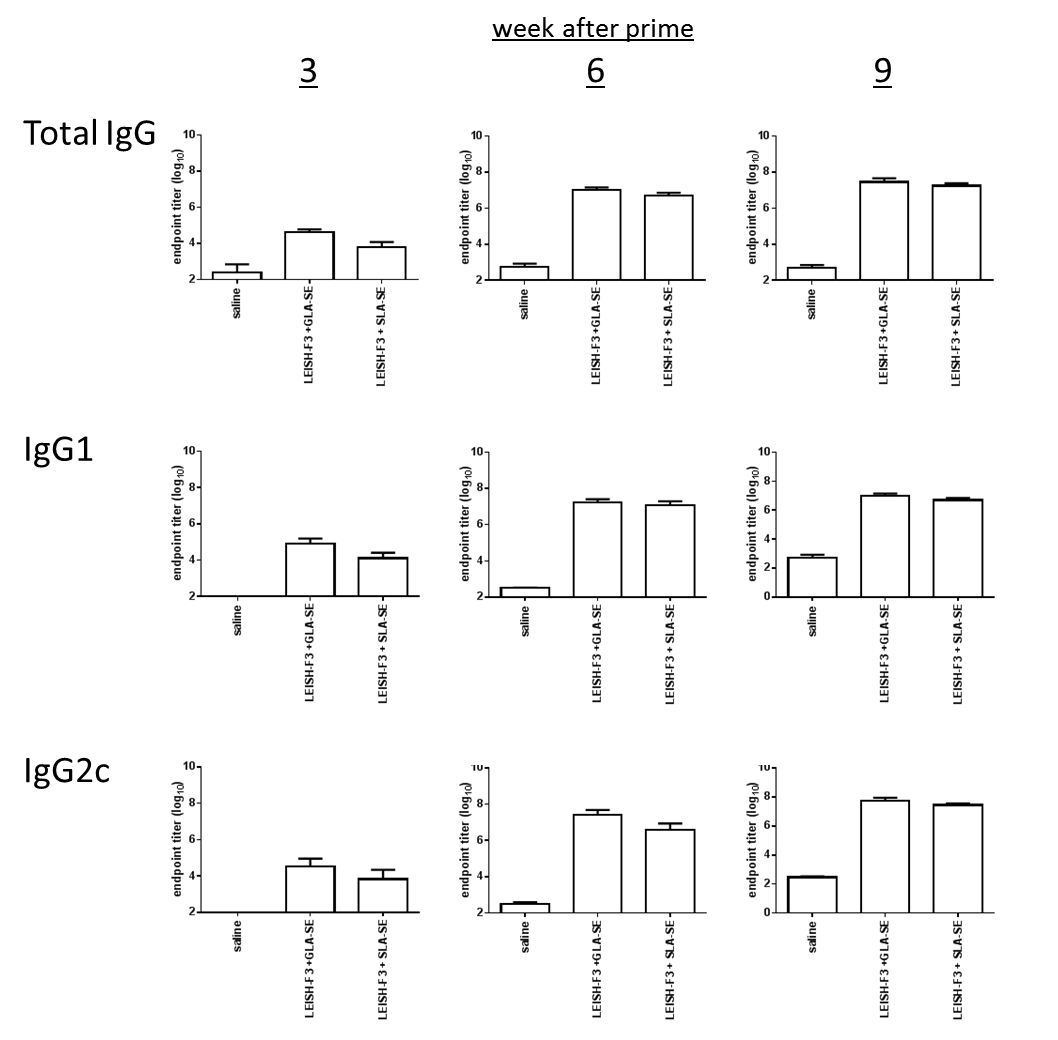
**
